# Pharmacoeconomic evaluation of weight-based vs fixed-dose pembrolizumab for non-small cell lung cancer in China

**DOI:** 10.3389/fimmu.2025.1665469

**Published:** 2025-10-15

**Authors:** Meiyu Wu, Yangke Yi, Andong Li, Kehui Meng, Zixuan Zhang, Chaohao Shi, Chongqing Tan, Xiaomin Wan, Jinan Ma

**Affiliations:** ^1^ Department of Pharmacy, The Second Xiangya Hospital, Central South University, Changsha, Hunan, China; ^2^ Institute of Clinical Pharmacy, Central South University, Changsha, Hunan, China; ^3^ Department of Oncology, The Second Xiangya Hospital, Central South University, Changsha, Hunan, China

**Keywords:** pembrolizumab, pharmacoeconomics, weight-based dosing, non-small cell lung cancer, China

## Abstract

**Background:**

While the immune checkpoint inhibitor pembrolizumab has improved outcomes for non-small cell lung cancer (NSCLC), the leading cause of cancer death in China, its standard fixed-dose regimen is costly. Given that studies demonstrate comparable efficacy between fixed and weight-based dosing, we aimed to compare the economic impact of these two dosing regimens in China.

**Methods:**

We conducted a one-year budget impact analysis from the Chinese payer perspective comparing fixed-dose (200 mg Q3W) versus weight-based (2 mg/kg Q3W) pembrolizumab regimens. Model parameters, including lung cancer epidemiology, treatment costs and market penetration rates, were derived from published studies and open-access databases. Scenarios with and without institutional vial dose-sharing were analyzed, and comprehensive one-way and probabilistic sensitivity analyses (PSA) were performed to assess model robustness.

**Results:**

The standard fixed-dose regimen was projected to have an annual cost of $6.67 billion. In a dose-sharing scenario, the weight-based regimen reduced annual drug consumption by 34.41%, resulting in a total saving of $2.29 billion. This lowered the annual cost per patient from $86,392 to $56,661. Without dose-sharing, the weight-based regimen still yielded an annual saving of $76.93 million. PSA confirmed the robustness of these findings, showing a 50% probability of achieving savings greater than $2.09 billion in the dose-sharing scenario.

**Conclusion:**

Adopting a weight-based dosing strategy for pembrolizumab, especially when optimized with vial-sharing protocols, offers substantial and achievable annual cost savings for the Chinese healthcare system without compromising therapeutic efficacy. Therefore, this regimen should be considered as a potential first-line treatment option for patients with advanced NSCLC.

## 1 Introduction

The incidence and mortality rates of lung cancer are the highest among all types of malignant tumors in China (1). It was reported that the number of new lung cancer cases and deaths in China in 2022 were 1,060,584 and 733,291, respectively (2). Among them, non-small cell lung cancer (NSCLC) is the most common type, accounting for about 80% to 85% (3, 4).

In recent years, the introduction of immune checkpoint inhibitors (ICIs) has changed the therapeutic landscape of NSCLC, especially programmed death receptor-1 (PD-1) and its ligand (PD-L1) blockers (5, 6). Multiple PD-1/PD-L1 inhibitors have been shown to significantly improve overall survival (OS) and progression-free survival (PFS) in NSCLC patients (7–10), and have been recommended by national and international guidelines as first- or second-line standard treatment options for NSCLC (11–13). Despite the clinical benefits of these drugs, their high prices place a heavy financial burden on patients and the healthcare system. It has been shown that drug costs constitute a major part of the direct medical costs of lung cancer patients in China (14). Therefore, how to optimize the cost of treatment while ensuring efficacy has become one of the global concerns in lung cancer treatment.

Despite the clinical efficacy of PD-1/PD-L1 inhibitors, current dosing regimens tend to use a ‘one-size-fits-all’ approach, administering a uniform dose regardless of the patient’s body type or the drug’s pharmacokinetics. Pembrolizumab, for instance, is routinely given at a fixed dose of 200 mg every three weeks (Q3W)—a regimen adopted more for administrative simplicity than for pharmacologic necessity. Yet this convenience may come at the cost of precision, as ICIs differ from traditional cytotoxic chemotherapies. Their pharmacodynamic profile is characterized by receptor saturation at low concentrations and long-lasting receptor occupancy, suggesting that higher doses do not necessarily confer greater benefit. The initial Phase I trial of pembrolizumab evaluated the pharmacodynamics of multiple doses and showed no pharmacodynamic differences between alternative doses of 1, 3, or 10 mg/kg (15). Translational modeling of intratumor exposure demonstrated that a dose of 2 mg/kg Q3W was able to achieve effective intratumor drug exposure and target inhibition (15). Population pharmacokinetic (popPK) modeling analyses showed that the area under the drug concentration-time curve (AUC) distributions of 200 mg overlapped sufficiently with the 2 mg/kg dose (16).

Despite these findings, fixed-dose regimens remain the standard approach. While this shift has simplified clinical practice, it has also led to widespread overtreatment, particularly among populations with low average body weights. Given these considerations, there is growing international interest in re-evaluating weight-based dosing strategies to better align drug utilization with individual patient characteristics (17–19). Notably, de-escalation approaches—including dose personalization and optimized vial utilization—are being actively explored as viable strategies to enhance the value of cancer immunotherapy while maintaining therapeutic efficacy (20).

Given the relatively low average body weight among Chinese adults (69.6 kg for males and 59 kg for females) (21), a fixed 200 mg dose may lead to unnecessary drug overexposure and resource waste. This study therefore aims to evaluate the differences in total drug utilization and direct medication costs between fixed-dose and weight-based (2 mg/kg) pembrolizumab regimens in the treatment of Chinese adults with NSCLC, providing evidence to inform more personalized and cost-effective dosing strategies.

## 2 Methods

### 2.1 Overview

We conducted a budget impact analysis (BIA) from the social perspective of payers in China to evaluate the economic differences between fixed-dose (200 mg Q3W) and personalized-dose (2 mg/kg Q3W) pembrolizumab regimens for the treatment of NSCLC, in accordance with the guidelines for BIA recommended by the International Society for Pharmacoeconomics and Outcomes Research (ISPOR) (22).We first estimated the number of patients potentially treated with pembrolizumab per year, and subsequently simulated total drug consumption under both dosing strategies, and considered two different medication management scenarios for personalized dosing: (1) dose-sharing (to optimize vial utilization) and (2) no-dose-sharing (to account for potential waste). Finally, we calculated and compared the total costs associated with each dosing regimen. Model construction and data analysis were performed in the R software (R version 4.0.5; http://www.r-project.org).

### 2.2 Target population

Based on the drug insert of pembrolizumab, we set the target population as locally advanced or metastatic NSCLC patients with epidermal growth factor receptor (EGFR) mutation-negative and anaplastic lymphoma kinase (ALK) mutation-negative (23). According to the National Bureau of Statistics, the number of male and female in China in 2024 was 719.09 million and 689.19 million, respectively (24). The National Cancer Center reported that the incidence rate of lung cancer in China was 91.36 per 100,000 for males and 58.18 per 100,000 for females (2). Studies have reported that NSCLC accounts for 80-85% of lung cancer types, and we took the mean value of 82.5% in our baseline analysis (4, 25, 26). The percentage of patients with advanced or metastatic NSCLC was 70.00% (27). Furthermore, the proportion of EGFR mutation-negative and ALK-negative patients has been previously estimated to be 42.1% (28). Therefore, the number of patients with locally advanced or metastatic NSCLC in China who were negative for EGFR gene mutation and ALK was about 257,212 cases. All variables used in the model are listed in **Table 1**.

**Table 1:** Variables used in the model.

Variable	Baseline value	Lower	Upper	Source
Population sizes in 2024 (thousand)	Male: 719,090 Female: 689,190	–	–	(24)
Incidence of lung cancer in male (per 100,000)	91.36	77.656	105.064	(2)
Incidence of lung cancer in female (per 100,000)	58.18	49.453	66.907	(2)
Proportion of lung cancer patients with NSCLC (%)	82.5	0.8	0.85	(4, 25, 26)
Proportion of NSCLC patients with advanced or metastatic (%)	70	59.5	80.5	(27)
Proportion of NSCLC patients with EGFR gene mutation negativity and ALK negativity (%)	42.1	35.785	48.415	(28)
Market share of pembrolizumab (%)	30	25.5	34.5	(27)
Male weight (kg)	69.6	59.16	80.04	(21)
Female weight (kg)	59	50.15	67.85	(21)
Drug cost per mg, US$	24.92	21.182	28.658	(29, 30)

NSCLC, non-small cell lung cancer; EGFR, epidermal growth factor receptor; ALK, anaplastic lymphoma kinase.

### 2.3 Market share

The market share for pembrolizumab was obtained from a budget impact analysis study (27), which projected hospital pembrolizumab utilization rate for 2024–2028 using relevant data of 183 hospitals from January 2020-October 2022. The study showed that the market share of pembrolizumab monotherapy in 2025 was 30%.

### 2.4 Cost of pembrolizumab

Currently, the price of pembrolizumab in China is ¥17,918 per 100 mg, equivalent to $24.92 per mg based on 2025 exchange rates ($1 = ¥7.19) (29, 30). Based on multiple studies indicating that drug dosage did not show a dose/exposure-dependent relationship with the incidence of most adverse events (15–18, 31, 35), we assumed comparable safety profiles for the fixed-dose and weight-based pembrolizumab regimens. Therefore, differential costs associated with treatment-related adverse events were not considered in our primary analysis.

### 2.5 Dosage strategy simulation

We simulated two dosage strategies, a fixed-dose strategy in which 200 mg would be used for all patients, and a weight-based dosage strategy in which the drug would be administered at 2 mg/kg according to the patient’s weight. According to the China Population and Nutrition Report, the average weight in China is 69.6 kg for males and 59 kg for females (21).In addition, we simulated two scenarios for the weight-based dosage strategy. Dose-sharing scenarios, where assuming there is no time limit on sharing single-dose vials, hospitals can use the remaining dose of a single medication for other patients, and the remaining medication is never discarded. No-dose sharing scenario, where each drug is used for only one patient and the remaining dose is discarded.

### 2.6 Uncertainty analysis

We conducted one-way sensitivity analyses to evaluate the robustness of our model by varying each parameter within plausible ranges or ±15% of baseline values. Furthermore, we conducted a probabilistic sensitivity analysis (PSA) involving 1,000 Monte Carlo simulations to evaluate the joint uncertainty of all model parameters. Each parameter was sampled from its predefined probability distribution. The PSA results enabled quantification of the probability that weight-based dosing would yield cost savings compared to fixed-dose administration.

## 3 Results

Under the current standard-of-care fixed-dose regimen, the model projected a total annual pembrolizumab consumption of 267.50 million mg for the eligible NSCLC patient population in China. This resulted in a total annual expenditure of $6.67 billion, equating to an average cost of $86,392 per patient per year (**Table 2**).

**Table 2:** Results of baseline analysis.

	Fixed dose	Weight-based dose	
		Dose-sharing	No-dose sharing
Number of patients in model	77,164	77,164	77,164
Annual dose consumption (million)	267.50	175.44	264.41
Average dose per patient (mg)	3,467	2,274	3,427
Annual Cost ($, million)	6,666.34	4,372.17	6,589.41
Annual cost per capita ($)	86,392	56,661	85,395
Annual Savings vs.Fixed Dose ($, million)		$2,294.17	$76.93

A shift to a weight-based (2 mg/kg) dosing regimen, when combined with optimal vial dose-sharing, was projected to yield substantial economic advantages. This strategy reduced the annual drug consumption by 34.41% to 175.44 million mg, culminating in a total annual cost of $4,372.17 million. This represents a remarkable annual saving of $2.29 billion compared to the fixed-dose regimen.

The critical importance of vial management was highlighted when analyzing the no-dose-sharing scenario. Although this approach still generated an annual saving of $76.93 million, the economic benefit was drastically reduced. This finding underscores that optimized institutional vial utilization is essential to realizing the full financial potential of weight-based dosing.

**Figure 1:**
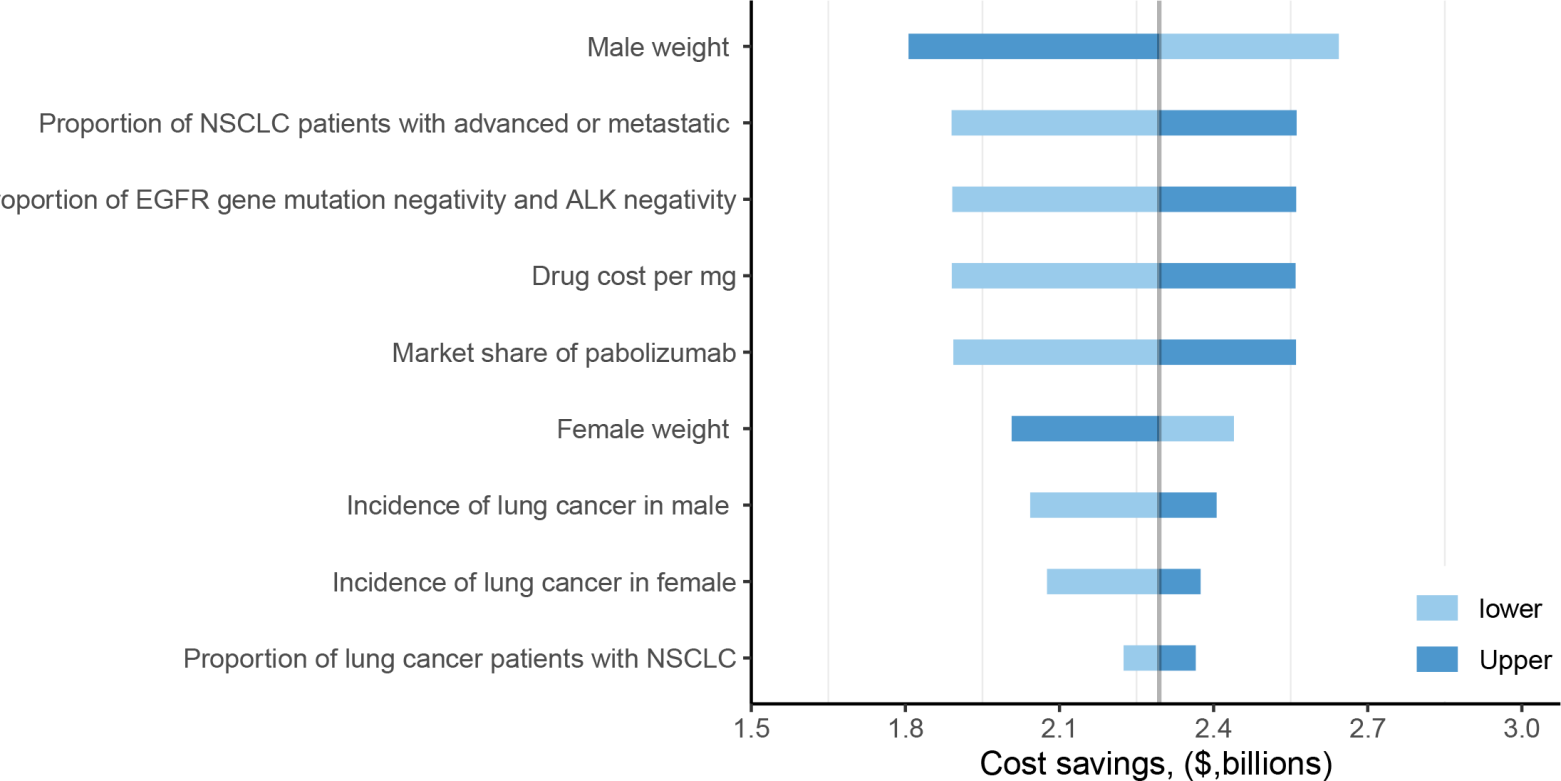
One-way sensitivity analysis of fixed dose versus weight-based dose in dose-sharing scenarios. NSCLC, non-small cell lung cancer; EGFR, epidermal growth factor receptor; ALK, anaplastic lymphoma kinase.

**Figure 2:**
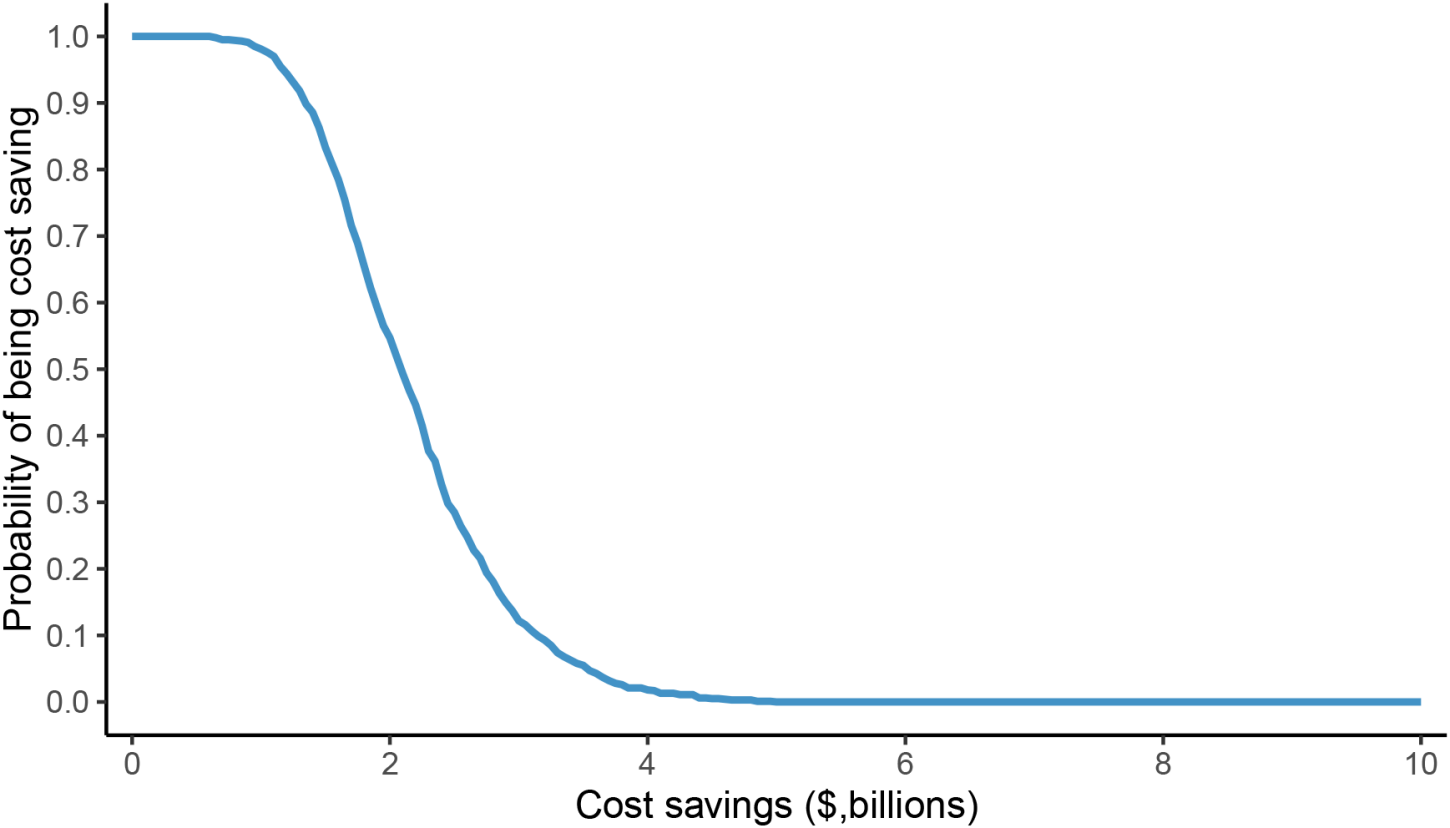
Probability sensitivity analysis of potential cost savings of fixed dose versus weight-based dose in dose-sharing scenario.

The one-way sensitivity analysis identified male body weight as the most influential parameter driving the economic outcomes. Variations in this single variable could alter the projected annual savings in the dose-sharing scenario by approximately $830 million, with savings ranging from $1.81 billion to $2.64 billion (**Figure 1**). The overall robustness of these findings was confirmed through probabilistic sensitivity analysis, which indicated a 50% probability that the cost savings would exceed $2.09 billion (**Figure 2**), reinforcing the significant and consistent economic advantage of the weight-based approach.

## 4 Discussion

Our study compared the economic benefits of using pembrolizumab at a fixed dose versus a weight-based dose for patients with advanced NSCLC in China. The results indicate that from the social perspective of payers in China, the 2 mg/kg strategy, especially when combined with dose-sharing, was expected to reduce the annual consumption of pembrolizumab from 267.5 million mg in the fixed-dose scenario to 175.44 million mg, resulting in annual cost savings of approximately $2,294 million. The weight-based program showed economic benefits even when doses were not shared. While the annual drug consumption of the regimen (264.41 million mg) was close to the fixed regimen, it could result in annual cost savings of $76.93 million.

Pembrolizumab was initially studied and approved at a weight-based dose (2 mg/kg) (32). The subsequent shift to a fixed-dose regimen, driven by manufacturers and regulators, has been the subject of discussion (32). This shift may have been influenced by economics, as fixed-dose regimens often result in higher average medication doses, generating more revenue for manufacturers, particularly following the withdrawal of small-size bottles from the market (33). Although fixed doses simplify clinical practice by eliminating the need to consider a patient’s weight, this approach can result in widespread overdose issues, leading to significant healthcare costs (34). Indeed, weight-based or body surface area-adjusted dosing has been successfully implemented for many cancer drugs over the past decades, which demonstrates that the healthcare system is capable of handling individualised dose calculations. Our study suggested the reintroduction or wider implementation of a cost-effective weight-based dosing strategy.

Notably, the substantial difference in cost savings between the dose-sharing and no-dose sharing scenarios ($2294.17million vs $76.93 million) highlights the importance of optimising vial utilisation to realise the economic benefits of weight-based dosing. Weight-based dosing requires exact dose adjustments according to patient weight, often yielding quantities that do not match whole vial increments. Currently, pembrolizumab is commonly packaged in 100 mg/vial, and the average weight in China is about 60–70 kg, which tends to lead to wastage of medicines when there is no dosage sharing or smaller-sized vials are not accessible, weakening the economic advantages of weight-based dosing. Although the operation may be slightly more complex, the economic and resource management benefits of implementing vial sharing would be substantial. It is suggested that policymakers and healthcare providers should establish standardized dose-sharing protocols and promote diverse vial sizes to maximize the cost-effectiveness of weight-based dosing strategies.

A key assumption of our model is the comparable safety profile between weight-based and fixed-dosing regimens, which warrants careful consideration. Our analysis did not account for potential differences in adverse event costs, a simplification based on the substantial body of evidence suggesting that pembrolizumab’s adverse event profile is not strongly dose-dependent within the clinically evaluated range. For instance, early pivotal trials like KEYNOTE-006, which compared 10 mg/kg regimens with different frequencies, did not report a significant increase in toxicity with higher dose intensity. Furthermore, extensive pharmacokinetic and exposure-response analyses have indicated that PD-1 receptor saturation occurs at relatively low drug concentrations, leading to a flat exposure-safety relationship for most common adverse events (35, 36). Modeling studies have also supported this observation, suggesting that adverse event rates are unlikely to vary significantly across approved dosing schedules (31). However, it is crucial to acknowledge the nuances of these findings. While receptor saturation may explain the lack of a steep dose-toxicity curve for on-target adverse events, it does not entirely rule out the possibility that higher drug exposure could increase the risk of specific or off-target toxicities. This remains an area of ongoing investigation.

Our study found significant cost savings with weight-based dosing for Pembrolizumab, similar to the results of several other studies. In Australia, a study showed that weight-based dosing of pembrolizumab would reduce drug acquisition costs by 23.5%, saving $467,996 in one year at one center (32). A modeling study in the United States showed that the use of weight-based dosing of pembrolizumab combined with vial sharing would reduce expenditures by 18% and save $3.6 million per year (37). In addition, a study from France noted that the use of fixed-dose therapy would result in a 26% increase in ICI costs (38). The convergent findings from diverse healthcare systems and patient populations collectively reinforce weight-based dosing as a cost-saving strategy with global relevance. This analysis focuses on comparing fixed-dose and weight-based dosing regimens. In practice, several modeling and clinical studies have explored alternative dosing strategies, including de-escalation approaches, personalized regimens, and hybrid models that combine weight-based and fixed dosing (39, 40). For example, Michiel M. Smeenk et al. conducted a large retrospective cohort analysis of NSCLC patients receiving pembrolizumab plus chemotherapy (40). They demonstrated that a hybrid dosing regimen was associated with non-inferior OS compared to a fixed-dose regimen, while significantly reducing pembrolizumab consumption and associated costs.

Our study not only quantifies the economic advantages of personalized pembrolizumab dosing in China, but also reveals potential economic limitations of the current fixed-dose regimen for PD-1/PD-L1 inhibitors. In current clinical practice, the standard regimen for most PD-1/PD-L1 inhibitors remains fixed-dose. This approach stems from the simplified design of early clinical trials rather than optimal pharmacological choices. However, accumulating evidence demonstrates that PD-1/PD-L1 inhibitors achieve PD-1 receptor saturation at lower doses, with no clinically meaningful efficacy improvement at higher concentrations. Consequently, adopting weight-based dosing for these agents would likely yield substantial economic benefits while maintaining therapeutic efficacy.

Several limitations in our study must be acknowledged. Firstly, as with all model-based studies, the validity of the input parameters is crucial to the reliability of our results. Although the model incorporated data from published literature and databases, discrepancies may persist between these sources and the real world. Although sensitivity analyses were conducted to assess the influence of parameter uncertainty, variations in these parameters could still alter the projected magnitude of cost savings. Secondly, our analysis focused exclusively on the direct drug costs of pembrolizumab. Due to data limitations, we did not consider other direct medical costs (e.g. administration of the drug and monitoring) or indirect costs (e.g. loss of productivity). Including these additional cost components might provide a more comprehensive socio-economic perspective. Thirdly, the market share of pembrolizumab was derived from a published study and did not account for dynamic factors such as emerging drug competition. However, we conducted one-way sensitivity analysis to evaluate the potential impact of market share variations on projected cost savings. Finally, due to the lack of long-term efficacy data, our study did not evaluate how weight-based dosing regimens affect the persistence of efficacy, recurrence risk, or the development of secondary resistance. These factors are critical for confirming the long-term clinical value and sustainability of this cost-saving strategy. Future research should incorporate these endpoints to validate the sustainability of the dosing strategy.

## 5 Conclusions

Based on our study, weight-based dosing of pembrolizumab, particularly when combined with a dose-sharing programme, could result in significant cost savings of up to $2.29 billion per year in China compared with a fixed-dose regimen. Although there are challenges in implementation, particularly with regard to vial use and standardized dose-sharing systems, our findings support revisiting weight-based dosing as a globally relevant strategy to enhance resource allocation without compromising efficacy. Policymakers should prioritize the use of personalized dosing to maximize the economic advantages of medications.

## Data Availability

The original contributions presented in the study are included in the article/supplementary material. Further inquiries can be directed to the corresponding author.
